# Systemically Administered Homing Peptide Targets Dystrophic Lesions and Delivers Transforming Growth Factor-β (TGFβ) Inhibitor to Attenuate Murine Muscular Dystrophy Pathology

**DOI:** 10.3390/pharmaceutics13091506

**Published:** 2021-09-18

**Authors:** Aqsa Iqbal, Ulrike May, Stuart N. Prince, Tero A.H. Järvinen, Ahlke Heydemann

**Affiliations:** 1 Department of Physiology and Biophysics, University of Illinois at Chicago, Chicago, IL 60612, USA; aqsa.iqbal@nghs.com; 2 Faculty of Medicine and Health Technology, Tampere University, 33014 Tampere, Finland; ulrike.may@posteo.net (U.M.); stuart.prince@utu.fi (S.N.P.); tero.jarvinen@tuni.fi (T.A.H.J.); 3 Department of Orthopedics & Traumatology, Tampere University Hospital, 33520 Tampere, Finland; 4 Sanford Burnham Prebys Medical Discovery Institute, La Jolla, CA 92037, USA; 5 Center for Cardiovascular Research, University of Illinois at Chicago, Chicago, IL 60612, USA

**Keywords:** vascular homing peptide, muscular dystrophy, fibrosis, transforming growth factor-β1 (TGFβ1), decorin, mdx, proteoglycan, extracellular matrix, angiogenesis, cell penetrating peptide, inflammation

## Abstract

Muscular dystrophy is a progressively worsening and lethal disease, where accumulation of functionality-impairing fibrosis plays a key pathogenic role. Transforming growth factor-β1 (TGFβ1) is a central signaling molecule in the development of fibrosis in muscular dystrophic humans and mice. Inhibition of TGFβ1 has proven beneficial in mouse models of muscular dystrophy, but the global strategies of TGFβ1 inhibition produce significant detrimental side effects. Here, we investigated whether murine muscular dystrophy lesion-specific inhibition of TGFβ1 signaling by the targeted delivery of therapeutic decorin (a natural TGFβ inhibitor) by a vascular homing peptide CAR (CARSKNKDC) would reduce skeletal muscle fibrosis and pathology and increase functional characteristics of skeletal muscle. We demonstrate that CAR peptide homes to dystrophic lesions with specificity in two muscular dystrophy models. Recombinant fusion protein consisting of CAR peptide and decorin homes selectively to sites of skeletal muscle damage in mdxDBA2/J and gamma-sarcoglycan deficient DBA2/J mice. This targeted delivery reduced TGFβ1 signaling as demonstrated by reduced nuclear pSMAD staining. Three weeks of targeted decorin treatment decreased both membrane permeability and fibrosis and improved skeletal muscle function in comparison to control treatments in the mdxD2 mice. These results show that selective delivery of decorin to the sites of skeletal muscle damage attenuates the progression of murine muscular dystrophy.

## 1. Introduction

Muscular dystrophies (MD) are the most prevalent genetic diseases in the world [1]. MDs are comprised of over 20 different genetic disorders, which result in progressive breakdown of skeletal muscle. They are characterized by membrane permeability, immune infiltration, myofiber necrosis, repeated rounds of degeneration and regeneration, eventual failure of the regeneration process, myofibroblast replacement of muscle tissue, and, finally, functionally impeding fibrosis [2,3]. Among the large numbers of different MDs, the most prevalent and one of the most severe is Duchenne MD (DMD), which can be modeled by mdx-mice.

TGFβ1 is the growth factor causative for the fibrotic aspect of many pathologies [4]. TGFβ1 is pathologically upregulated in human and murine MD [5]. In addition, TGFβ1 inhibition reduces murine MD-mediated fibrosis and immune system-mediated pathogenesis [6]. mdx mice treated with TGFβ1 neutralizing antibody for six weeks had reduced fibrosis, decreased immune infiltration, improved respiratory functions, and enhanced fore-limb grip strength [7]. However, because of the embryonic lethality of the TGFβ1 knockout mouse [8], it is understood that a full global absence of TGFβ1 is inconsistent with health [9]. Therefore, skeletal muscle-specific targeted therapies need to be considered [6,10,11].

Two of the major problems encountered using systemic drug delivery is that only a small fraction of the administered drug reaches its desired location in the body and side effects are encountered elsewhere in the body [12,13,14,15,16]. This last has been the major obstacle for translating potent TGFβ inhibitors into clinical practice as the generalized inhibition of TGFβ has triggered autoimmunity in the human body [8]. For these reasons, not only specificity on its molecular target, but also target organ selectivity are desired from any potential drug candidate. Target organ specificity for any systemically administered drug can be obtained by exploiting a natural platform that exists in the tissues, organotypic vasculature [12,13,14,15,16]. Each organ places unique molecular structures, “fingerprints”, in its blood vessels, essentially creating a postal code system, “vascular zip codes”, within the vasculature of our bodies [12,13,14,15,16]. Moreover, diseases induce the expression of disease-specific molecular signatures on their vasculature. These disease-associated blood vessels are structurally distinct, on the molecular level, from the rest of the blood vessels in the body, and thus serve as an appealing target for organ-specific delivery of systemically administered therapeutics in regenerative medicine [12,13,14,15,16].

We have previously identified a vascular homing peptide for the targeted delivery of systemically administered therapeutics to focal tissue injury sites [16]. The vascular homing peptide CAR (sequence CARSKNKDC) was originally identified as it recognizes angiogenic blood vessels in regenerating tissues (skin wounds and tendon ruptures/lacerations) [16]. More recently, we and others have established that CAR peptide also homes to inflammation foci in diseases such as pulmonary arterial hypertension, myocardial infarction, and aortal aneurysm [17,18,19,20,21,22,23,24,25,26,27]. In addition to being a specific homing peptide, the CAR peptide is also a cell penetrating peptide capable of delivering pharmaceutical agents (even large nanoparticles) to target organ parenchyma [17,18,19,20,21,22,23,24,25,26,27]. We have previously generated a recombinant multi-functional fusion protein, CAR-DCN, where CAR peptide is used as a delivery vehicle for the therapeutic protein decorin (DCN) [25]. DCN is a small leucine-rich proteoglycan that is a naturally occurring TGFβ inhibitor [28,29,30], but also inhibits other important inducers of scar formation such as connective tissue growth factor (CTGF/CNN2) [31], epidermal growth factor receptor (EGFR/ErbB1), and the rest of the ErbB-receptors, ErbB2-4 [32], Met receptor [33] as well as another member of TGFβ superfamily, myostatin [34]. The cell attachment afforded by the CAR peptide in CAR-DCN has substantially increased DCN’s biological activity against TGFβ over native DCN and systemically administrated CAR-DCN accumulated in healing skin wounds in significantly larger quantities than native DCN [25]. These features were associated with more rapid wound healing [25], suppressed scar formation [25], and attenuated abdominal aortic aneurysm progression [19].

We show in this study that CAR peptide has a unique homing specificity toward damaged skeletal muscle foci in mdxD2 mice and in gamma-sarcoglycan mutated DBA2/J mice (SgcgD2), while it does not target normal skeletal muscle. Furthermore, CAR-DCN accumulates in the skeletal muscles of mdxD2 mice, and it suppresses fibrosis better than the native DCN or DCN delivered with mutant CAR peptide (*m*CAR-DCN). The decreased fibrosis was significant in the canonical skeletal muscle groups (quadriceps and hamstring muscles). The CAR-DCN treatment also decreased membrane permeability in the abdominal, quadriceps, and hamstring muscle groups. Furthermore, functional analysis revealed that CAR-DCN treatment significantly improved skeletal muscle function in murine MD.

## 2. Materials and Methods

### 2.1. Peptide Production

Peptides were synthesized with an automated peptide synthesizer by using standard solid-phase fluorenylmethoxycarbonyl chemistry. During synthesis, the peptides were labeled with fluorescein isothiocyanate (FITC) using an amino-hexanoic acid spacer as described previously [35,36]. We have previously shown that replacing two basic amino acids in the CAR sequence with neutral ones (CA***R***S***K***NKDC mutated to CA***Q***S***N***NKDC in *m*CAR) eliminates the wound and inflammation homing activity [16,17,25].

### 2.2. Recombinant Protein Production

Recombinant DCN, CAR-DCN, and *m*CAR-DCN proteins were expressed in 293-F cells using the FreeStyle 293 expression system (Thermo Fisher Scientific, Waltham, MA, USA) as described previously [25,36]. Briefly, a pcDNA3.1/myc-HIS-C plasmid encoding for either DCN, CAR-DCN, or *m*CAR-DCN was mixed with Opti-MEM I media and 293fectin transfection reagent. The DNA:293fectin solution was added to 293-F cells and the cells were cultured for 48 h. Recombinant protein was isolated from the media utilizing the C-terminal 6XHistidine tag for binding onto Ni-NTA agarose beads (Qiagen) using 5 mL of beads per 500 mL of media. After an overnight incubation at 4 °C, the beads were washed with PBS, and CAR-DCN was eluted with PBS containing 300 mM imidazole, dialyzed against PBS, and stored at −80 °C. All peptides and recombinant proteins used in the experiments are summarized in Table 1.

### 2.3. Characterization of Recombinant Decorins

Recombinant DCNs were analyzed on SDS/PAGE on 4–20% acrylamide gradient gels. The gels were either stained with Coomassie Blue or used to transfer the proteins to a PVDF membrane and immunoblots were performed either with mouse monoclonal anti–6-histidine tag antibody (clone 18,184; Novus Biologicals) or mouse monoclonal anti-human DCN (MAB143, R&D Systems) antibodies. The primary antibodies were detected by goat anti-mouse IgG–HRP (Bio-Rad) and then developed using ECL plus a chemiluminescence reagent (Amersham Biosciences).

### 2.4. Heparan Sulfate Binding Analysis

The binding ability of recombinant CAR-tagged proteins (CAR-DCN) to heparan sulfate at 25 °C were analyzed via Octet biosensor interferometry on the Octet RED384 System (ForteBio, Fremont, CA, USA) in a 96-well plate format. Heparan sulfate (fast moving fraction sodium salt from porcine mucosa, Sigma-Aldrich H9902) was coupled to Octet AR2G (Amine Reactive 2nd Generation; ForteBio) biosensors via ADHZ and nontoxic picoline borane complex-based reductive amination. The heparan sulfate immobilization protocol was related to that published by Cochran et al. [37] for heparin immobilization onto surface plasmon resonance BIAcore sensor chips. An AR2G sensor was first activated in 0.2 M EDC/0.05 M NHS (Sigma-Aldrich, St. Louis, MI, USA) mixture (600 s, 100 rpm) to enable amine coupling to 100 mg/mL ADHZ (Sigma-Aldrich; 600 s, 100 rpm). Following a brief wash, ADHZ was then joined to the C-terminal carboxyl group of heparan sulfate via reductive amination using 1 mg/mL heparan sulfate supplemented with 3.75% acetic acid and 0.25 M picoline borane complex (Sigma-Aldrich; diluted 1:10 from 2.5 M frozen stock in 100% DMSO; 2 × 1800 s, 100 rpm). Finally, after a brief wash, the unreacted amine sites were quenched in 1 M ethanolamine (Sigma-Aldrich; 600 s, 100 rpm) before thorough washing. The control/reference sensor was coupled to 0.02 M lactose instead of heparan sulfate using the same chemistry.

For binding analysis of proteins (DCN-CAR, DCN), the sensor shake speed was 1000 rpm. A baseline was recorded for 180 s, association of protein (500, 250, 125, 62.5, and 31.3 nM) to heparan sulfate were performed for 600 s, followed by dissociation for 600 s. The sensors were regenerated with 4 M NaCl at the end of each cycle.

The data were recorded and analyzed via Octet Data Acquisition and Analysis Software 7.0 (ForteBio). Lactose sensor data were subtracted from the heparan sulfate sensor data (reference sensor subtraction), the *Y*-axis was aligned to baseline, and interstep correction was calculated to association.

### 2.5. Animals

ɣ-Sarcoglycan null mice on the DBA2/J (S*gcg*D2) background were obtained from Elizabeth McNally and *mdx*DBA2/J (*mdx*D2) were obtained from Jax Labs (stock number D13141) and then kept in a specific pathogen free facility at the University of Illinois at Chicago. All mouse protocols performed in this work adhered to the Guide for the Care and Use of Laboratory Animals (National Institutes of Health) and the protocol of the institutional animal care and use committee of the University of Illinois at Chicago. In addition, the ARRIVE guidelines were adhered to. Mouse numbers were pre-chosen to include a minimum of six mice per trial group. In all studies, the mice were randomized between the various treatment and control groups. In addition, male and female mice were equally distributed. The investigators were blinded to the treatments. This number was chosen from previous, preclinical muscular dystrophy treatment publications [38].

### 2.6. Peptide Targeting

The three-week old mdxD2 mice received a 100 µL bolus injection of FITC-labeled CAR or *m*CAR peptide at (40 µg/100 µL PBS, approximately 2 µg/g), directly into their right quadriceps muscles and 100 µL saline was injected into the contra-lateral muscle. The three-week old SgcgD2 mice received 40 µg/100 µL of FITC-labeled CAR or *m*CAR peptide intravenously. The mice were perfused and euthanized 2 h later and their quadriceps were flash-frozen for direct immunofluorescent visualization of FITC-labeled peptides.

### 2.7. Treatment Schedules

Protein or vehicle intra-peritoneal 100 µL injections of both male and female (from both mutant mouse lines) were assigned to one of four groups: CAR-DCN 40 µg/100 µL PBS (*n* = 16), *m*CAR-DCN 40 µg/100 µL PBS (*n* = 9), DCN 40 µg/100 µL (*n* = 11), or PBS (*n* = 6). The treatment dose was based on previous treatment trials [19,25]. All mice were injected three times a week for three weeks beginning at three-weeks old. We did not adjust the dose to animal weight as the animals were all of similar weights and the males and females were assorted evenly to the four groups. We also did not adjust the dose for molarity, however, the CAR-DCN and *m*CAR-DCN groups received less substance then the control DCN group, so this oversight did not impact our results toward a false positive effect. The mice were not injected with peptide or PBS on their last day. Nineteen days after the injections began, the mice were analyzed by whole-body plethysmography and then injected with 10 mg/g of Evans blue dye (EBD) in PBS. At euthanasia, the gastrocnemius with the soleus muscle were immediately ex vivo analyzed on the Aurora muscle test system (Aurora, Ontario) for strength and fatigue parameters. The other muscles were harvested for histology, immunofluorescence, Evans blue dye (EBD) uptake, or hydroxyproline content (HOP).

### 2.8. Immunofluorescence

Immunofluorescence (IF) was conducted as described previously [38]. Liquid nitrogen cooled isopentane frozen muscle tissue sections were cut 10 µm thick on a cryostat. The sections were fixed in ice cold methanol for 5 min, rinsed 3 × 3 min in PBS, blocked in 5% FBS, 1% Tween-20 in PBS for 15 min, and incubated with primary antibodies overnight at 4 °C. The primary antibodies were anti-HIS (Santa Cruz at 1:50), anti-p-SMAD2/3 (Santa Cruz at 1:50), dystrophin (Dys2, Novocastra Labs at 1:100), or anti- α-smooth muscle actin (Sigma Chemicals at 1:100). After 3 × 15 min washes, the sections were incubated with species-specific secondary, 488 nm wavelength antibodies for 1 h at room temperature. After three more 15 min washes, the slides were mounted with DAPI and coverslips. Background staining controls were conducted on serial sections by eliminating the primary antibody. These sections displayed no immunogenic staining (data not shown). Pictures were acquired on an Axiophot, with identical exposure settings within each stain on all of the tissues.

### 2.9. Histology

A subset of quadriceps muscles were harvested and immediately placed in 10% neutral buffered formalin. The muscles were sent to University of Illinois at Urbana-Champaign veterinary clinic for paraffin processing, cutting, and Masson’s Trichrome staining. The pictures were captured on an Axio Imager Z2 at an original magnification of 20×.

### 2.10. Evans Blue Dye (EBD) Uptake

The EBD uptake assay to measure membrane permeability, as an early indicator of MD disease progression, was performed as described previously [38]. The tissues destined for EBD uptake quantification were minced and weighed into a pre-tared Eppendorf tubes at harvest. The tubes were immediately frozen in dry ice. On the assay day, 500 µL of formamide was added to each tube, which was then placed into a 55 °C water bath for at least 2 h with periodic shaking. A total of 200 µL of the formamide with eluted EBD was then transferred to a 96-well plate and optical density was measured at λ = 580 nm. A standard curve was also created, and the results are reported as nM/mg tissue. The muscle EBD uptake values were normalized to that animal’s kidney values, which were not statistically different between the treatment groups, but allowed for normalization of intra-peritoneal injection efficiency.

### 2.11. Hydroxyproline (HOP) Assay

The HOP assay was performed as previously reported, with some modifications [38]. The tissues destined for the HOP assay were minced and weighed in a pre-tared Eppendorf tube and immediately frozen in dry ice. On the assay day, 100 µL of 6 M HCl was added to each tube and the solutions were boiled overnight at 104 °C. On the following day, 10 µL of hydrozylate was placed in a fresh tube with 150 µL isopropanol, and 75 µL of a 7% Chloramine T solution, then the solution was mixed and incubated at room temperature for 10 min. Next, 1 mL of Ehrlich’s reagent (0.3% p-dimethylaminobenzaldehyde in ethanol with 6.75% sulfuric acid, mixed 3:13 with isopropanol) was added, the solution was mixed and incubated in a 65 °C water bath for 30 min. The samples were immediately quenched in ice, followed by transferring 200 µL to a 96-well plate and taking the optical density at λ = 620 nm. A standard curve was also created and the results reported as nM/mg tissue.

### 2.12. Gastrocnemius and Soleus Muscle Function Testing

Because the animals were six weeks old at analysis, the extensor digital longus muscles (the usually analyzed muscle in the MD field) were too small for analysis. Instead, the gastrocnemius/soleus muscles were tied off at the Achilles tendon and the sciatic nerve with 4.0 suture and quickly mounted onto the test system in Krebs–Henseleit buffer, with a slow stream of room air. The optimal length was determined starting at a resting tension of 5 g using the sine wave function. This was followed by three percent-strain relax tests, one tetanus test and three mixed tests. Because we could not obtain a consistent electrical response from the muscles, we report the lengthening contraction responses and the electrical contraction responses (when achieved) separately.

### 2.13. Plethysmography

We used the Buxco (Data Science International, St. Paul, MN, USA) whole body plethysmography device as per the manufacturer’s instructions. On the day of the procedure, the mice were acclimatized to the chambers for 10 min and then four 15 min recordings were conducted. Each animal was analyzed in all four chambers to identify any possible chamber disparities and provide 60 min of data for each animal. The four chamber values were compared for each mouse and if required, data from a chamber was discarded if it was greater than one standard deviation away from the average of the other three chambers. All of the data for the suspect chamber was discarded if this situation arose (two fifteen-minute recordings, from different animals, were discarded for inconsistency). We expect that this chamber disparity occurred due to insufficient closing of the chamber.

### 2.14. Statistics

Data are shown as mean averages with S.E.M. One-way analyses of variance with Tukey post-hoc test was utilized for all comparisons (StatView). *p* values < 0.05 were considered statistically significant.

## 3. Results

### 3.1. CAR Peptide Targets Muscular Dystrophy Lesions in Skeletal Muscle

**Figure 1:**
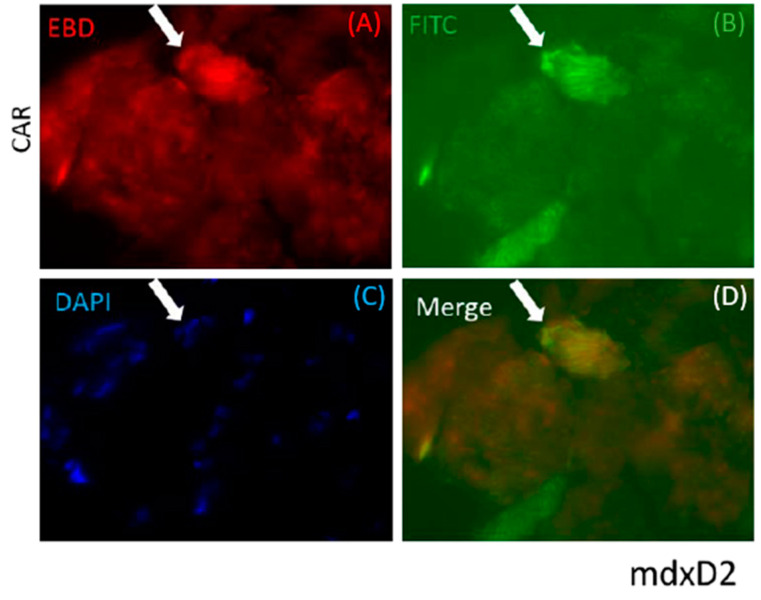
The CAR peptide co-localized to damaged (indicated by EBD staining) muscle cells in the mdxD2 mice. The quadriceps of these mice were directly injected with CAR-FITC peptide and harvested 2 h later. The EBD positive fiber demonstrated co-localization with FITC coupled CAR peptide (arrow). Slides were fluorescently visualized with: (**A**) EBD for membrane permeability, (**B**) directly for the FITC peptide, (**C**) DAPI for the nuclei, and (**D**) merged images. Original magnification was 40×.

**Figure 2:**
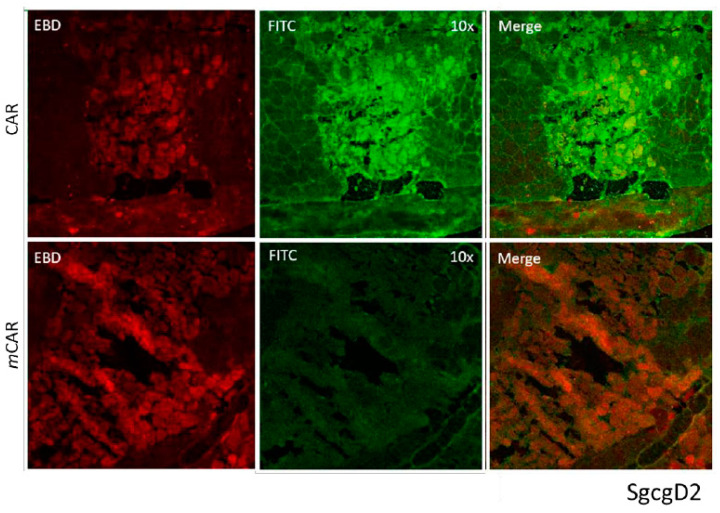
The CAR peptide co-localized to damaged (indicated by EBD staining) muscle cells in the SgcgD2 mice. These mice were injected IV with either the CAR-FITC or the mCAR-FITC peptide. The EBD positive fibers (red) demonstrated co-localization with FITC coupled CAR peptide (green). The mCAR peptide (FITC coupled) did not co-localize to the damaged, EBD positive fibers. Original magnification was 10×.

CAR peptide was originally identified as a peptide capable of targeting angiogenic vasculature forming at the site of tissue injury [16,25]. However, recent research has shown that it also targets damaged vasculature in inflammatory diseases [17,18,19]. This prompted us to explore whether CAR peptide could home to dystrophic lesions in mouse models of muscular dystrophy. FITC-labeled CAR peptide or mutated CAR peptide (*m*CAR; CA***R***S***K***NKDC mutated to CA***Q***S***N***NKDC, which abolishes the homing activity almost completely [16,17,25] were injected into two different muscular dystrophy models to determine the peptide homing capabilities to dystrophic muscle lesions. In the mdxD2 model, CAR or *m*CAR tagged with FITC was directly injected into the left quadriceps muscle, the contralateral muscle received PBS. The quadriceps were harvested 2 h post injection, flash frozen, cryosectioned, and directly visualized. The FITC-tag from the chimeric protein co-localized with EBD positive fibers in mdxD2 mice (Figure 1). The ScgcD2-mediated MD mice received intra-venous, systemic injections of either CAR or *m*CAR peptides, both FITC-labeled. The quadriceps were harvested 2 h post-injection, flash frozen, cryosectioned, and directly visualized. In these mice, the CAR peptide also co-localized with damaged regions of dystrophic muscles (top panel, Figure 2), while no co-localization was seen with the *m*CAR peptide (bottom panel, Figure 2). These results indicate that locally and systemically administered CAR sequence specifically targeted damaged lesions in two models of dystrophic muscles and specifically delivered the FITC cargo to these lesions.

### 3.2. Decorin Fused to CAR Peptide Maintains Its Biological Activity

We then expressed and purified recombinant DCN proteins: native decorin (DCN), decorin targeted with CAR peptide (CAR-DCN), and decorin targeted with mutated CAR peptide (*m*CAR-DCN). All versions contained a C-terminal 6XHis-tag for purification and detection. We have previously characterized the recombinant DCN proteins in detail [19,25]. Now, we first confirmed the quality of the expression and purification by testing the recombinant DCN proteins in SDS gel electrophoresis, where the recombinant proteins migrated at the correct size; sharp bands at 45 kDa with a smear above the band (Figure S1). The sharp bands correspond to the core DCN proteins, and the smear was caused by heterogeneity in the chondroitin sulfate chain attached to the DCN molecules (Figure S1). All recombinant proteins were detected by anti-6-histidine tag antibody (Figure S1). Next, we explored whether the CAR peptide expressed within a large recombinant protein retains its biological function. As CAR is a heparin binding peptide and has a heparan sulfate binding function [16,25], we used this ability to create a novel high-throughput Octet biosensor assay to measure the interaction of our CAR-tagged proteins to heparan sulfate. CAR-DCN binds to the heparan sulfate sensor, but does not bind to a sensor coated with lactose (negative control). In contrast, the native DCN did not bind to heparan sulfate (Figure S1).

### 3.3. CAR-DCN Homes to Muscular Dystrophy Lesions

Next, we assessed the homing capabilities of the recombinant CAR-DCN protein. DCN itself has been reported to home to “activated” vasculature through a core protein-dependent interaction [39,40]. Due to these homing features, DCN or peptides derived from it have been used as a delivery vehicle for other therapeutics [41]. Furthermore, the GAG side chain of DCN can bind to integrins on sprouting endothelial cells, possibly further enhancing the homing capability of the DCN [42]. Our recombinant DCNs have a naturally occurring GAG side chain attached to the core protein ([25], Figure S1).

**Figure 3:**
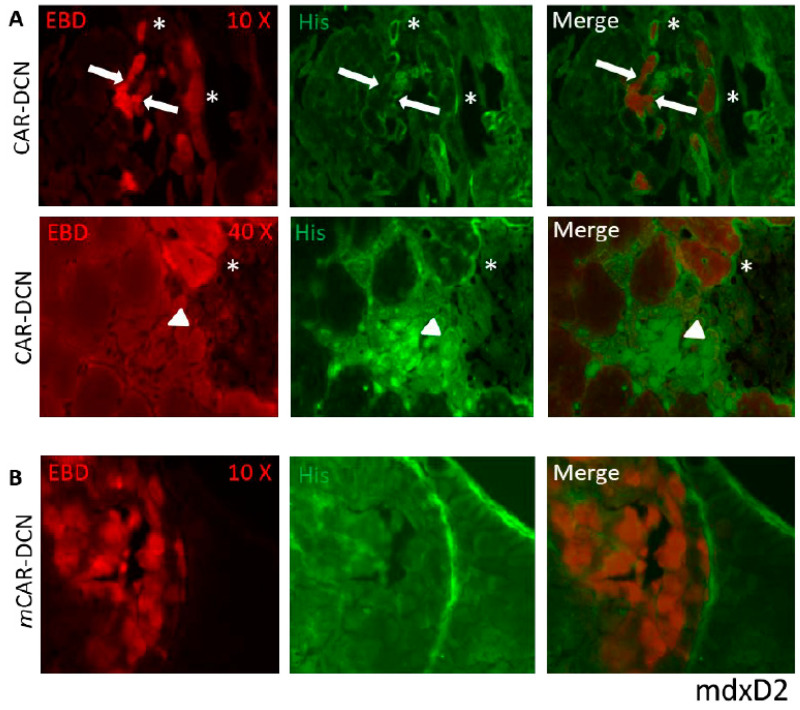
CAR-DCN localizes to sites of dystrophin deficient muscle damage. Staining of quadriceps muscles from mdxD2 mice injected IP, for three weeks with CAR-DCN or *m*CAR-DCN (in which the CAR homing peptide was mutated) reveals CAR targeted to sites of muscle damage by fluorescence microscopy. Slides were visualized with EBD for membrane permeability (red, first column) and anti-histidine antibodies for the recombinant proteins (green, middle column), and last column shows merged images. (**A**) The top row indicates co-localization of the CAR-DCN to the sarcolemma of damaged (red) cells and surrounding extracellular matrix (asterisks in top two rows). However, not all damaged cells have attracted the peptide (white arrows). The second row is a higher magnification illustrating CAR-DCN homing to regenerating skeletal muscle cells, which are negative for EBD and have likely resealed their membranes (arrow heads). (**B**) The quadriceps from mdxD2 mice were injected with *m*CAR-DCN. No co-localization of the peptide with damaged cells, in any pattern, was present. Original magnification for (**A**) panel top row and (**B**) panel was 10× and for (**A**) panel second row 40×.

**Figure 4:**
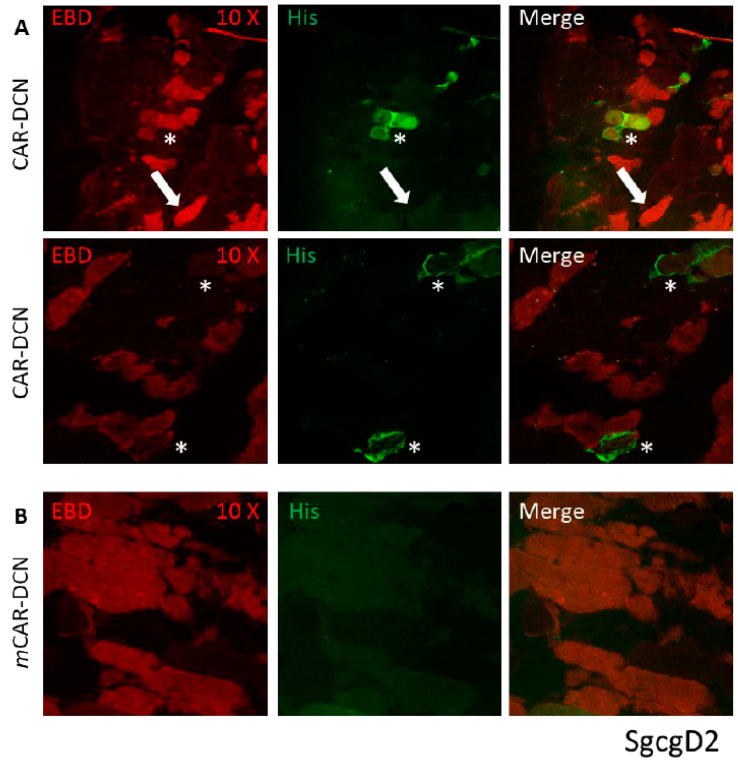
CAR-DCN localized to damaged muscle cells in the ɣ-sarcoglycan null quadriceps muscles, a second model of muscular dystrophy. (**A**) Similar to the mdx model injected CAR-DCN co-localized to a subset of damaged muscle tissues. The sgcgD2 mice received three weeks of IP injections every other day. Slides were visualized with EBD for membrane permeability (red, first column) and anti-histidine antibodies for the recombinant proteins (green, middle column), and last column shows merged images. In many EBD positive fibers, CAR has targeted to the sarcolemma (asterisks, top and middle rows). In other EBD positive fibers, no His-tag staining for the recombinant protein was detected (arrows). (**B**) The DCN protein containing mutant CAR (mCAR-DCN) never co-localized to damaged cells. Original magnification was 10×.

Mice from both muscular dystrophy models were IP injected on alternate days for three weeks testing recombinant DCN proteins expressed together with both peptides: CAR and *m*CAR. Both mdxD2- (Figure 3) and SgcgD2-mediated MD (Figure 4) demonstrated specific targeting of DCN by CAR to the dystrophic lesions. The damaged skeletal muscle cells stained red with Evans blue dye (EBD), and the homing was identified via anti-HIS antibodies and fluorescence microscopy. CAR-DCN homed specifically to the damaged regions, often co-localizing with the EBD signal while the dystrophic lesions were negative for the *m*CAR-DCN control protein. In addition, normal skeletal muscle remained negative for CAR-DCN homing (Figure S2).

Of interest are the differing levels and patterns of CAR-DCN homing demonstrated in different models of muscular dystrophy. Many EBD positive (which identifies pathologically permeable membranes) fibers had strong CAR-DCN homing to the sarcolemmal membrane (*, Figure 3 and Figure 4), while other EBD positive fibers contained no detectable levels of CAR fusion protein (some examples identified with arrows, Figure 3 and Figure 4). We also saw in the mdx tissue, but not in the SgcgD2 muscles analyzed, robust targeting of CAR-DCN to regenerating fibers in a peri-nuclear pattern (arrow heads, Figure 3). This finding is in line with the CAR peptide’s cell penetrating activity [16,25].

### 3.4. Treatment of Muscular Dystrophy with Recombinant DCNs

Encouraged by the specific homing of CAR-DCN to the regions of dystrophic muscle damage, we wanted to assess the therapeutic value of CAR-DCN against muscular dystrophy. The dose of recombinant DCNs administered was chosen on the basis of previous CAR-DCN studies [19,25]. The mdxD2 animals were treated with either DCN, CAR-DCN, *m*CAR-DCN, or PBS. The mice were monitored daily during the treatment trial and no side effects (such as lack of grooming or malaise) were observed. The animals’ weights were measured at the beginning and at treatment completion. No differences in their weights were observed (Figure S3), indicating that none of the treatments impacted the normal growth within male or female animal groups. Except for differences in weight between male and female mice, there were no further differences between the genders, therefore, both genders were combined in all subsequent analyzes.

### 3.5. Targeted CAR-DCN Reduces TGFβ1 Signaling in Muscular Dystrophy

**Figure 5:**
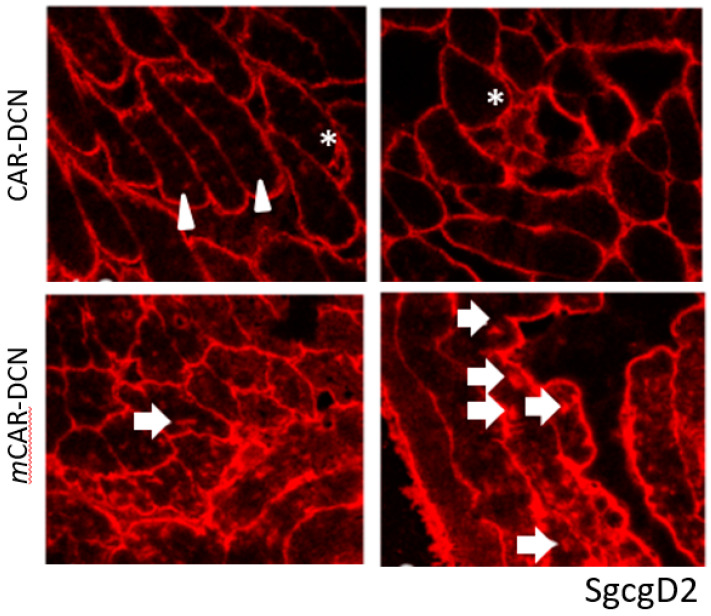
Three-weeks of CAR-DCN treatment decreased p-SMAD2/3 staining in the SgcgD2 mouse model. p-SMAD2/3 specific antibodies followed by species specific secondary antibody were used to detect nuclear and therefore activated p-SMAD2/3. Although these animals were not injected with EBD (to reserve the robust red channel for low-abundance p-SMAD staining), the damaged fibers can be identified because of the variable fiber size (*) and central nuclei (arrowheads). All panels are from SgcgD2 quadriceps injected with either CAR-DCN or *m*CAR-DCN. Original magnification 40×.

We then investigated whether CAR-DCN could inhibit TGFβ1 signaling in MD mice. After the three-week intraperitoneal (IP) treatment course of CAR-DCN, quadriceps muscles from SgcgD2 mice were analyzed for p-SMAD2/3 immuno-staining (Figure 5). Intra-nuclear p-SMAD2/3 staining indicates active TGFβ1 signaling. Mice receiving the mutant CAR-DCN protein (*m*CAR-DCN) had more p-SMAD2/3 positive nuclei than the CAR-DCN receiving animals. CAR-DCN also decreased p-SAMD2/3, as shown via immuno-fluorescent staining in the mdxD2 mouse model of MD (Figure S4). The decreased nuclear staining of p-SMAD2/3 after injection of CAR-DCN demonstrated that CAR-DCN inhibited TGFβ signaling (Figure 5, top row).

### 3.6. Targeted CAR-DCN Reduces α-Smooth Muscle Actin (αSMA) Staining in Muscular Dystrophy

**Figure 6:**
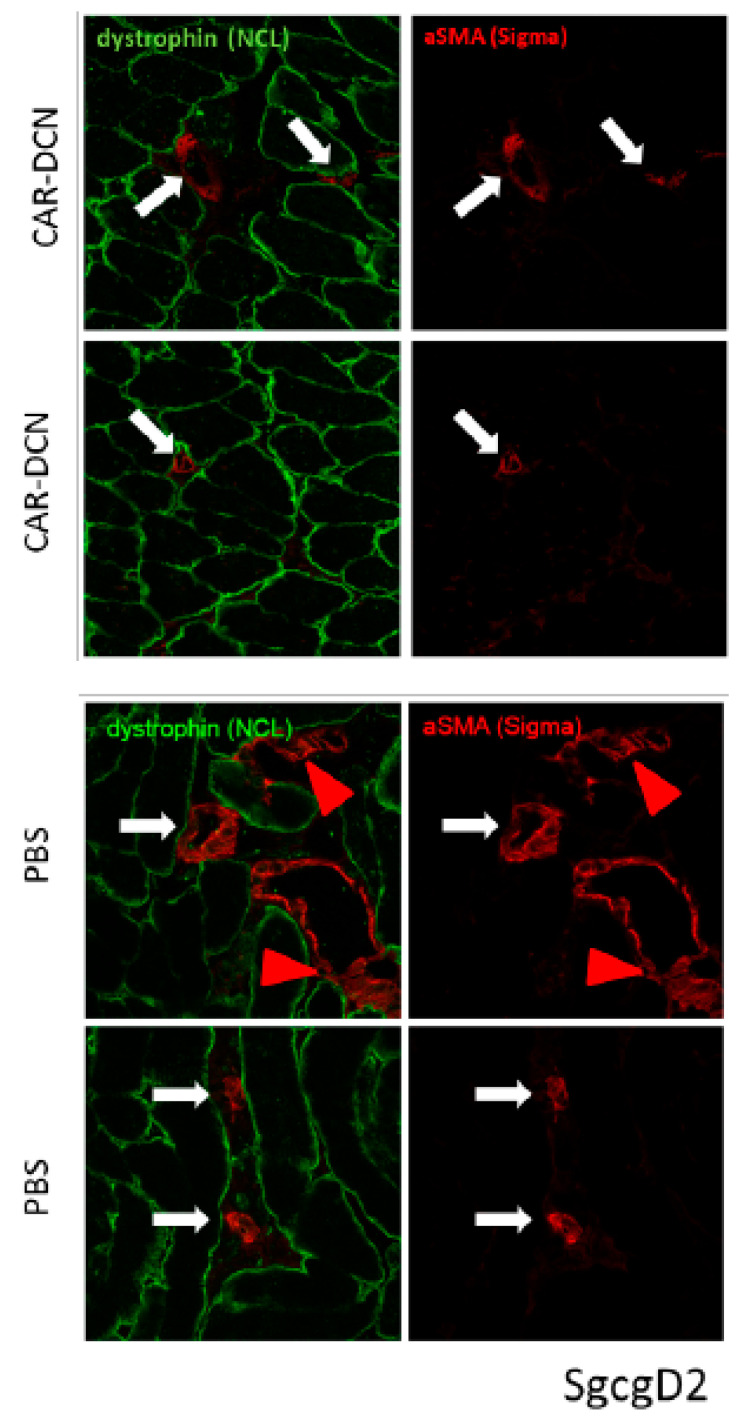
Three weeks of CAR-DCN treatment resulted in decreases in α smooth muscle actin staining in quadriceps muscle. SgcgD2 quadriceps were stained with dystrophin and α smooth muscle actin. The blood vessels from CAR-DCN treated mice had staining for α smooth muscle actin (white arrows, top four panels), but did not have extravascular staining (i.e., myofibroblasts). The PBS control quadriceps had both vessel staining (white arrows) and extracellular staining (red arrowheads, bottom four panels) indicating myofibroblast transformation. In addition, the dystrophin staining appeared more uniform in the CAR-DCN treated mice. Original magnification 40×.

Myofibroblasts are contraction capable cells transformed from normal fibroblasts and are responsible for fibrosis formation in different diseases [43]. TGFβ signaling is needed to convert normal fibroblasts to myofibroblasts and the resultant myofibroblasts can be detected with *α*SMA staining [43]. Three weeks of CAR-DCN treatments also reduced αSMA actin staining in the SgcgD2 mouse line. Quadriceps muscle tissues were stained with dystrophin and αSMA specific antibodies. The treated mice demonstrated more uniform dystrophin staining and reduced non-vessel αSMA (Figure 6).

### 3.7. Skeletal Muscle Morphology Is Improved by CAR-DCN Treatment in Muscular Dystrophy

**Figure 7:**
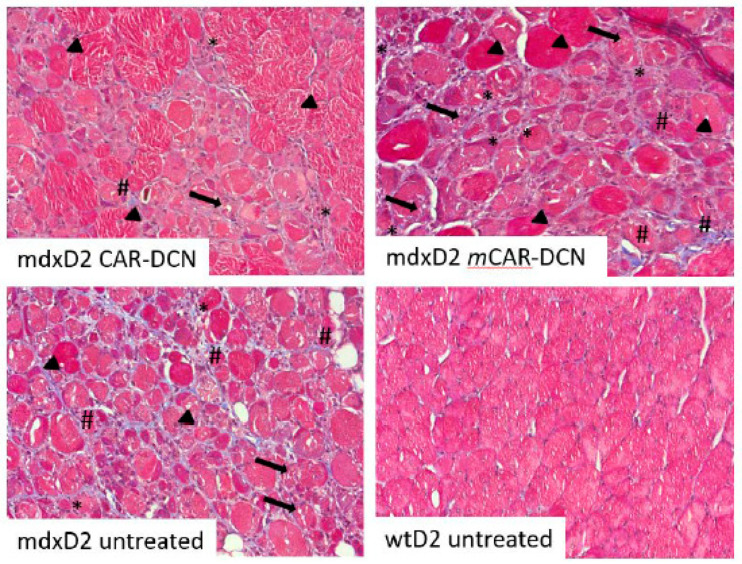
More robust regeneration after CAR-DCN treatment of mdxD2 mice. These representative Masson’s Trichrome pictures are from CAR-DCN, mCAR-DCN, or control three week treated mice. The vastus lateralis of the quadriceps was imaged at original magnification of 20×. Late stage regenerative fibers, identified by central nuclei and almost normal size (arrowheads); necrotic fibers are the cells with many gaps (arrows); immune infiltrate identified with closely packed nuclei (asterisks); and fibrotic areas identified by the blue staining (number sign) are indicated in the images. The mCAR-DCN treated muscle had more pathology as revealed by fewer normally sized muscles, more immune cells between muscle cells than in the CAR-DCN treated mice, and the distinctive presence of fibrotic areas. Original magnification 10×.

After three weeks of treatment, histological analysis demonstrated less muscle pathology and enhanced regeneration in the vastus lateralis of the quadriceps in the CAR-DCN group than in the *m*CAR-DCN group in mdxD2 mice (representative pictures, Figure 7). CAR-DCN treated mdxD2 quadriceps had regenerating fibers with central nuclei, some degenerating fibers with necrotic portions as well as some infiltrating immune cells. MdxD2 mice treated with *m*CAR-DCN had the same cells, but in different proportions than in CAR-DCN treated mice; the degenerative fibers were more numerous and large areas of interstitial fibrosis were also more prevalent in the *m*CAR-DCN treated muscles than in the CAR-DCN treated. Overall, larger proportions of the CAR-DCN treated muscle cells appeared to be at the finishing steps of regeneration because the fibers were more uniform in size, they lacked interstitial fibrosis and had a reduced number of immune cells infiltrating between the muscle cells than the muscles treated with *m*CAR-DCN.

### 3.8. Pathological Sarcolemmal Permeability Is Significantly Reduced by CAR-DCN Treatment

**Figure 8:**
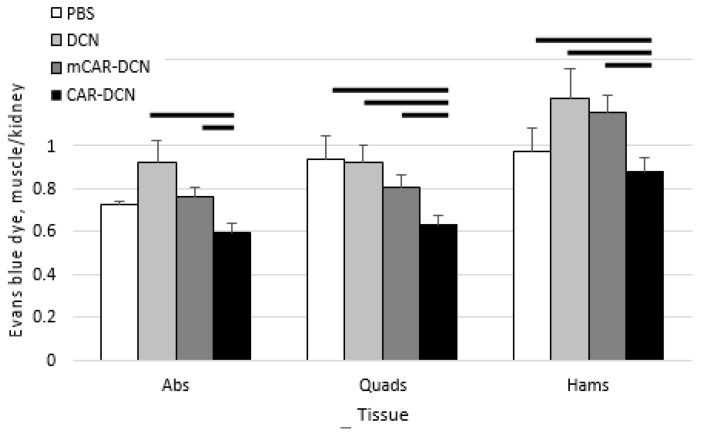
CAR-DCN treatment of mdxD2 mice significantly decreased membrane permeability in the two muscle types analyzed. Three week-old mdxD2 mice were treated with CAR-DCN, decorin (DCN), mutant CAR-DCN (*m*CAR-DCN), or PBS for a period of three weeks, and were then assessed by skeletal muscle Evans blue dye (EBD) uptake normalized to kidney EBD uptake. Abs, abdominals; Quads, quadriceps; Hams, hamstring. Data represent mean ± SEM. Solid lines indicate statistical significance (*p* < 0.05). *n* = 7, 11, 10, 10 animals, respectively.

Because CAR-DCN treated quadriceps muscles showed substantially larger areas of regenerating tissue in histological analysis, we decided to perform quantitative assays to verify the treatment outcomes. Pathological sarcolemmal permeability is a feature of MD in both mdx mice and patients. It is well-documented that excessive TGFβ signaling increases this permeability [44]. Therefore, we assessed sarcolemmal permeability by measuring the amount of cellular internalized EBD in the different treatment groups. The analysis of EBD demonstrated that CAR-DCN treatment reduced the membrane leakage significantly in the studied muscles (quadriceps, hamstrings) and trended to decrease in the abdominal muscles, whereas the other treatments did not reduce pathological sarcolemmal permeability (Figure 8). These results demonstrate that CAR-DCN reduced TGFβ-driven pathologic sarcolemmal permeability in murine MD.

### 3.9. Muscular Collagen Deposition Is Decreased with CAR-DCN Treatment

**Figure 9:**
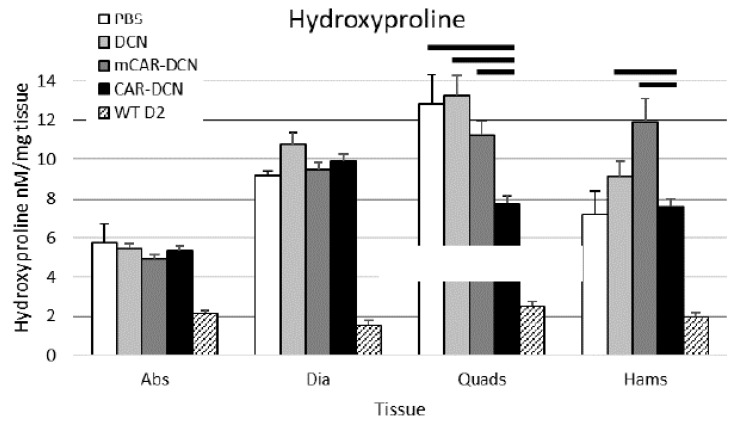
Three weeks of CAR-DCN treatment decreased fibrosis as measured by the hydroxyproline content assay in the canonical skeletal muscle tissues. Abs, abdominals; Dia, diaphragm; Quads, quadriceps; Hams, hamstring. Data represent mean ± SEM. Solid lines indicate statistical significance (*p* < 0.05). *n* = 7, 11, 10, 10 animals, respectively.

The histological evaluation already indicated that CAR-DCN treatment was able to reduce the fibrotic deposits in MD. Next, we quantitatively investigated whether CAR-DCN treatment reduced fibrosis in the mdxD2 animals. Hydroxyproline levels were determined from different skeletal muscles as a quantitative measure of fibrosis. Fibrosis was significantly reduced by CAR-DCN treatment in the analyzed skeletal muscle groups (quadriceps and hamstrings, Figure 9). These data are consistent with the histological staining of the quadriceps muscles, which showed a substantial reduction in the amount of fibrotic tissue by CAR-DCN treatment (representative pictures, Figure 7). The other control treatment groups did not show any reduction in the HOP levels in any of the muscle groups analyzed. The non-canonical skeletal muscle tissues (abdominal and diaphragm muscles) did not exhibit decreased fibrosis by CAR-DCN treatment. Concerning fibrosis in heart, the inhibition of fibrosis improves cardiac function in MD [44]. None of the mdxD2 mouse hearts had hydroxyproline levels significantly greater than the wild type controls at this young age; the hearts of MD mice were not yet damaged by MD (six weeks old, Figure S5).

### 3.10. CAR-DCN Treatment Enhances Skeletal Muscle Function

**Figure 10:**
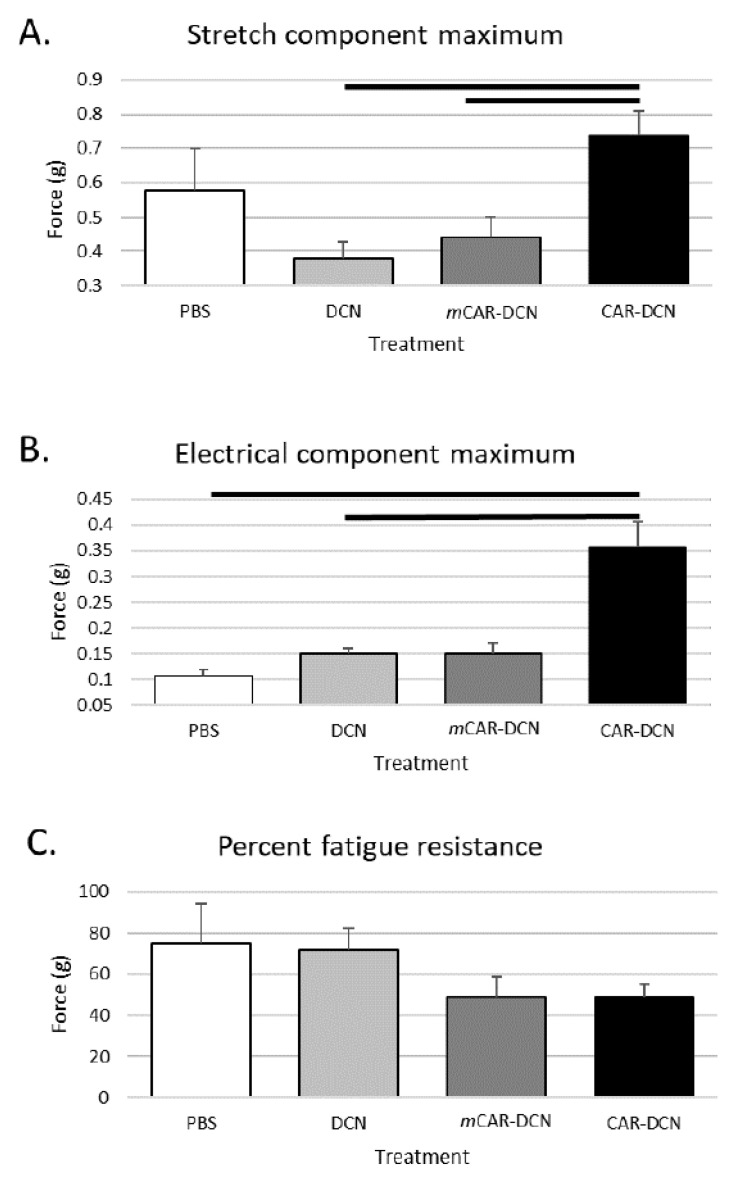
Gastrocnemius/soleus muscle tissues of mdxD2 mice treated with CAR-DCN for three weeks are able to produce more force than muscle tissues of the control mdxD2 mice. (**A**) The stretch component test indicated significant force improvement in the CAR-DCN muscles. (**B**) The increased force was also demonstrated when assessed by the electrical component of the test. (**C**) However, mdxD2 mice treated with CAR-DCN did not show improvement in percent fatigue resistance. The solid lines indicate statistical significance (*p* < 0.05). *n* = 6, 11, 9, 10 animals, respectively.

We also assessed the gastrocnemius/soleus muscles with an ex vivo muscle function apparatus to determine the functional and mechanical properties of these skeletal muscles. The CAR-DCN treated muscles were significantly stronger by multiple metrics than the muscles from other treatment groups (Figure 10). The CAR-DCN treated muscles displayed significantly stronger contraction when stimulated by stretching or by electricity when compared to the other three treatment groups (Figure 10, top and middle graphs). However, the CAR-DCN group experienced similar muscle fatigue after three successive stretch-induced contractions compared to the control treatment groups (Figure 10, bottom panel).

### 3.11. No Respiratory Defect Is Detected in Young Muscular Dystrophy Mice

Prior to euthanasia, all of the mice were tested with the Buxco plethysmography apparatus because late-stage MD causes respiratory deficits. None of the treatment groups nor untreated mdxD2 mice were significantly different for any of the respiratory parameters from wildtype control mice. The analyzed parameters include the important PENH parameter, which is often increased in older MD mouse models [45] due to the excessive diaphragm fibrosis (Figure S6). Furthermore, the inflammatory reaction developed faster in limb muscles than in respiratory muscles (diaphragm) in MD models [46], indicating that the respiratory deficit may take some time in these mice.

## 4. Discussion

We demonstrate the specific targeting of muscular dystrophy lesions by a systemically administered vascular homing peptide, CAR. To our knowledge, this is the first report of a technology that enables systemic administration of a pharmaceutical, yet muscle lesion-specific targeting in the treatment of life-threatening muscular dystrophies. We found that CAR peptide homes within skeletal muscle to the muscular dystrophy lesions, which will be primary targets of therapeutic MD interventions. We also show as a proof-of-principle experiment that we can target a potential therapeutic molecule, DCN, to these same lesions by the CAR peptide and that the targeted therapy is significantly better in suppressing the fibrosis formation than the non-targeted therapy in the treatment of MD. Thus, the CAR peptide-provided targeting of the muscular dystrophy lesions may offer entirely new opportunities to treat muscular dystrophy more effectively and with fewer side effects than previously anticipated.

We now demonstrate that the CAR peptide specifically homes to areas of degenerating and regenerating muscle cells. Interestingly the homing pattern of recombinant CAR-DCN fusion protein was more restricted within dystrophy lesion than the ubiquitous homing by CAR peptide. This may indicate that although CAR peptide is able to target muscular dystrophy lesions thoroughly, the therapeutic protein in the fusion molecule, DCN, defines some of its localization within the targeted area. This possibility is supported by the fact that DCN can bind to multiple extracellular matrix proteins and cell surface receptors such as integrins (for review please see [47]. Thus, one could theoretically deliver multiple therapeutic molecules simultaneously to the target site by CAR and then the therapeutic molecules could further specify their respective molecular targets within the damaged lesions. We can envision delivering pro vesicle fusion proteins such as Annexin 6 [48] to the cells in the early phases of muscle damage, DCN, to inhibit excessive TGFβ signaling to the cells undergoing regeneration, and hypertrophy inducing molecules such as MyoD during the final regeneration processes. This strategy would circumvent the usual issues associated with the asynchronicity of muscle regeneration in MD. The specific homing of CAR peptide in MD warrants future studies to test whether selective targeting of additional pharmaceuticals can be obtained in MD.

Mouse models of the disease have proven invaluable in establishing a natural history of the disease [38] and for preclinical treatment trials. The mdx mouse on the standard C57Bl/10 background has a rather mild disease progression and does not resemble the severe disease phenotype of human DMD [49,50]. Therefore, a mdx mutant mouse on the DBA2/J background (mdxD2) was generated [51,52]. This mouse strain is highly fibrotic due to a polymorphism in latent transforming growth factor β (TGFβ) binding protein 4 (LTBP-4) [52], which causes excessive TGFβ signaling and severe fibrosis. The D2 background had the most severe MD disease compared to three other commonly used mouse strains [38], and therefore mdxD2 is considered as the most representative model of DMD [50,51]. Consequently, testing any potential therapy in the mdxD2 mice will provide a robust assessment of potential therapies that are able to alleviate DMD pathology, especially the functionally impeding fibrosis. In addition, the gamma-sarcoglycan mutation on the DBA2/J background (SgcgD2), a faithful model of Limb Girdle Muscular Dystrophy-2C, provides a second model to verify potential therapies. Excessive TGFβ signaling is documented in both the murine MD models and in DMD [5,53,54]. Due to the complex nature of TGFβ signaling, we utilized the proven method to assess the TGFβ signaling by determining whether p-SMAD2/3 is localized to the nucleus. In the untreated or control treated dystrophic muscles, the active p-SMAD2/3 signaling was present, while this staining was not detectable after the 3-week treatment course with CAR-DCN. TGFβ inhibition has proven beneficial in murine MD models: it reduces chronic inflammation, pathological membrane permeability, and fibrosis [52]. In current work, we verified that TGFβ inhibition reduced membrane permeability and fibrosis.

The precise mechanism whereby reduced TGFβ signaling reduces membrane permeability is not yet understood, despite the many publications supporting this causality [38,52]. It could be hypothesized that by inhibiting the chronic immune response, the pathological membrane permeability is also prevented.

The various muscle groups did not respond similarly to the CAR-DCN treatment in terms of fibrosis. Only the canonical skeletal muscles demonstrated decreased fibrosis after the CAR-DCN treatment. Although we have shown in our previous works that the phenotype in other muscle groups—abdominals and diaphragm—is improved with TGFβ1 inhibition [52], these other muscle groups may require higher doses or longer administration of CAR-DCN than in the current study or their vascular zip codes may be different from the one targeted by CAR. One additional possibility is that we used intra-peritoneal dosing instead of i.v.-injections as in previous CAR-DCN-studies. The uptake of CAR-DCN could be reduced from the intraperitoneal cavity as tissues outside the blood vessel lumen could express the “CAR receptor”. These possibilities need to be evaluated.

We also demonstrate increased muscle force production in response to stretch and electrical stimuli in the CAR-DCN treated than in the control groups. However, the CAR-DCN treatment did not affect the muscles’ resistance to fatigue. The treatment did not improve respiratory parameters or cardiac function, mainly due to the young age of the mice, who had not yet developed dysfunction.

DCN is a natural TGFβ inhibitor that has been shown to inhibit TGFβ-driven scar formation in numerous disease models including injured skeletal muscle [34,55]. It is especially a relevant therapeutic option for skeletal muscle and muscular dystrophy due to its biological functions outside of TGFβ inhibition. DCN also inhibits another member of the TGFβ-superfamily, myostatin [34]. Myostatin inhibits skeletal muscle regeneration after injury and causes fibrosis/scar formation in injured skeletal muscle [56] and the inhibition of myostatin improves the outcome in MD [57,58,59]. Furthermore, DCN is also a potent inhibitor of connective tissue growth factor (CTGF/CCN2) [31]. CCN2 is over-expressed in different MDs [60] and its activity level correlates with the extent of fibrosis in muscular dystrophies [61]. The virus-mediated overexpression of CNN2 in normal skeletal muscle induces a phenotype that resembles MD [62]. As the binding sites for TGFβ, myostatin and CCN2 reside in different parts of DCN, a single DCN molecule could simultaneously block three mediators of fibrosis in MD. In addition to the inhibitory functions on the growth factors that induce scar formation in MD, DCN is classified as a myokine as it is secreted during muscle contractions and plays a role in muscle growth [63,64]. Furthermore, DCN is also a potent anti-inflammatory molecule [47,65]. Dampening of chronic inflammation can reduce fibrosis and muscle fiber damage in MD [2].

We have previously shown that CAR-DCN is substantially more active in suppressing TGFβ1 than native DCN in vitro where the target-specific homing does not play a role [25]. CAR peptide is a heparin and heparan sulfate binding peptide that anchors CAR-DCN on the cell surface [25]. This effect may enhance its biological activity and provide unique selectivity by bringing it to the proximity of heparin binding and scar-inducing isoforms of TGFβ, namely TGFβ1 and -β2 [66,67]. Interestingly, large number of naturally occurring TGFβ/bone morphogenetic protein (BMP) antagonists are also heparin binding proteins [67]). Thus, CAR peptide may have converted DCN into a selective inhibitor of scar-inducing isoforms of TGFβ [25]. The re-engineering of the therapeutic proteins by providing them with additional heparin binding domain could be an emerging theme in tissue engineering as a similar approach to one used in the engineering of CAR-DCN has been utilized more recently in generating “super” growth factors [68]. The “super” growth factors were created by fusing a growth factor with a heparin binding domain from placental growth factor (PLGF). Whereas the CAR peptide has homology with the heparin binding domain of BMP4 [16], the addition of the heparin binding domain of PLGF makes the chimeric, “super” growth factors more active than the native ones by affording them enhanced heparan sulfate-dependent cell binding and presentation to the growth factor receptors [68].

DCN has high structural homology to another member of the small leucine-rich repeat proteoglycan (SLRP) family, biglycan (BGN). BGN has been used to treat experimental MD models with great success [69]. BGN mitigates the phenotype of mdx mice as it can recruit utrophin, a dystrophin paralog, to the dystrophin glycoprotein complex (DGC) and rescue the biomechanical defect associated with the loss of dystrophin [70]. To recruit utrophin, BGN needs to be expressed devoid of its two glycosaminoglycan (GAG) side chains (i.e., as a core protein) to be able to bind to extracellular members of DGC [71]. BGN´s binding site to the sarcoglycans—DGC members—is in the N-terminus. DCN does not share this binding site [69]. This raises a possibility for a generation of chimeric BGN-DCN molecule, where the N-terminus of DCN is replaced by one from BGN. This could potentially yield a superior therapeutic molecule; one with DGC binding activity from BGN and the above described anti-fibrotic and regenerative activity from DCN, while also being devoid of GAG as the only GAG binding site of DCN is in its N-terminus [72,73].

## 5. Conclusions

In this study, we showed the specific targeting of MD lesions by the systemically administered vascular homing peptide, CAR. We also demonstrated, as a proof-of-principle experiment, that we could target a potential therapeutic molecule, DCN, to these lesions by CAR peptide and the targeted therapy is significantly better in suppressing the fibrosis formation than the non-targeted DCN therapy in the treatment of MD. Thus, the CAR peptide-provided targeting of the MD lesions may offer entirely new opportunities to treat MD more effectively and with fewer side effects than previously anticipated.

## Figures and Tables

**Table 1:** The vascular homing peptides and recombinant proteins used. For targeting experiments, the FITC dye was coupled to the synthesized CAR and *m*CAR (mutant CAR peptide) peptides. For recombinant protein expression, the homing peptide CAR or mutant CAR (*m*CAR) was cloned to the C-terminal end of human decorin (DCN). An epitope tag of 6-histidines was also added for purification to the molecule following the CAR sequence. The recombinant proteins were expressed in mammalian expression system and purified by chromatography.

Name	Peptides & Recombinant Proteins
CAR	CARSKNKDC
*m*CAR	CAQSNNKDC
DCN	5′-hDCN—HHHHHH-3′
CAR-DCN	5′-hDCN—CARSKNKDC—HHHHHH-3′
*m*CAR-DCN	5′-hDCN—CAQSNNKDC—HHHHHH-3′

## Data Availability

The data presented in this study are available on request from the corresponding author.

## Supplementary Materials

The following are available online at https://www.mdpi.com/article/10.3390/pharmaceutics13091506/s1. Figure S1: Characterization of recombinant decorin proteins, Figure S2: CAR-DCN treatment did not cause protein localization in wild type skeletal muscle, Figure S3: None of the treatments affected the weight gain of the animals over the three-week treatment course, Figure S4: CAR-DCN reduces p-SMAD2/3 nuclear staining in mdxD2 quadriceps muscles, Figure S5: mdxD2 mice do not develop more fibrosis than wild-type control mice, Figure S6: CAR-DCN treatment does not affect respiratory status in muscular dystrophy mice. Figure S7: Uncropped western blot membrane and Coomassie-stained SDS/PAGE-gel.
