# Supplementary material for: Systemically Administered Homing Peptide Targets Dystrophic Lesions and Delivers Transforming Growth Factor-β (TGFβ) Inhibitor to Attenuate Murine Muscular Dystrophy Pathology
Source: Pharmaceutics. 2021 Sep 18;13(9):1506. doi: 10.3390/pharmaceutics13091506 (PMC8471674; doi:10.3390/pharmaceutics13091506)
Supplement: Supplementary file 1 [file pharmaceutics-13-01506-s001.zip › pharmaceutics-1362637-supplementary.pdf]

pharmaceutics

Supplementary Materials: Systemically administered homing peptide targets dystrophic lesions and delivers transforming growth factor-β (TGFβ) inhibitor to attenuate murine Muscular Dystrophy pathology

Aqsa Iqbal, Ulrike May, Stuart N. Prince, Tero A.H. Järvinen and Ahlke Heydemann

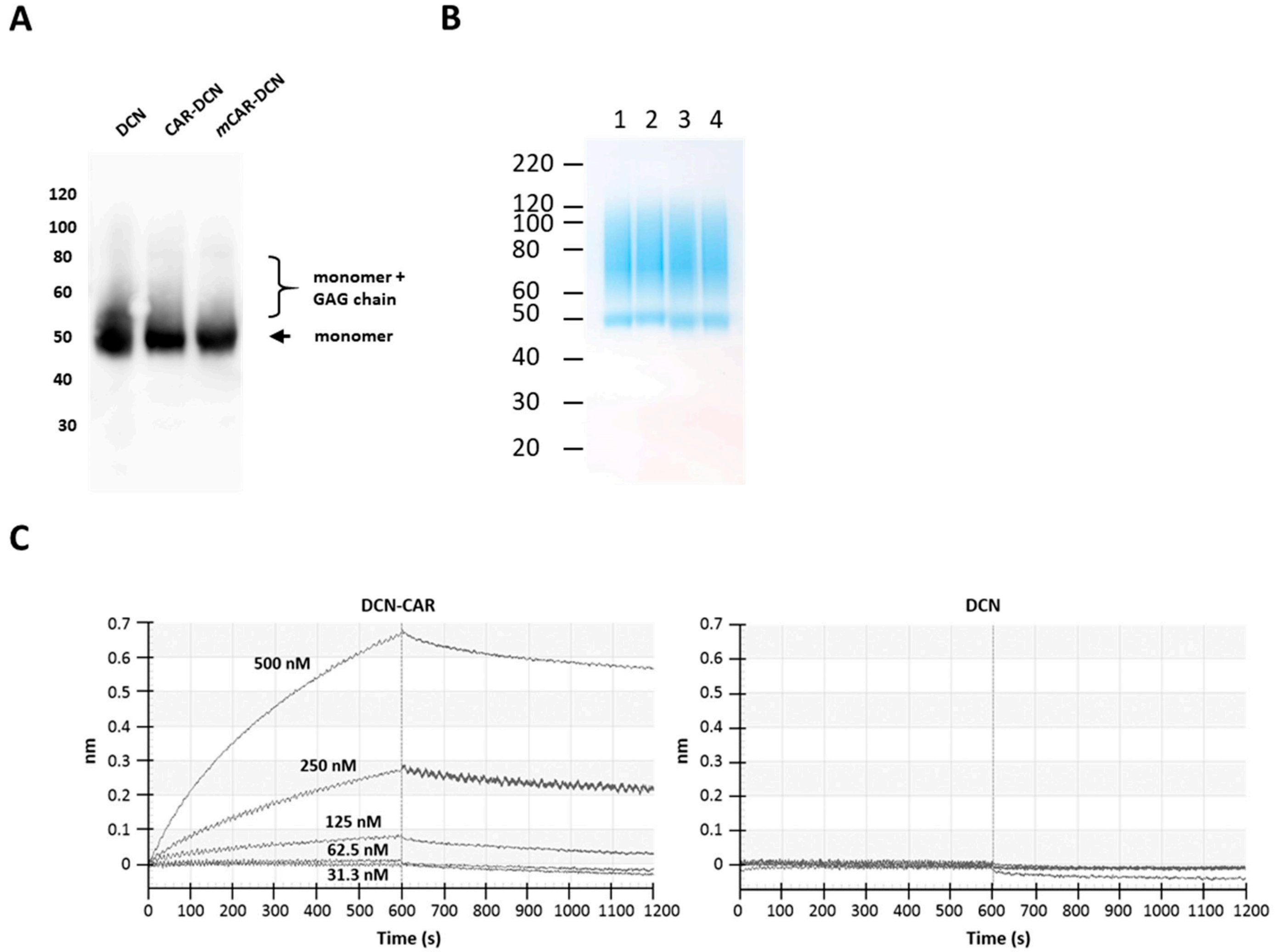

Figure S1. Characterization of recombinant decorin proteins. (A) Gel electrophoretic analysis of recombinant decorins (DCNs). The recombinant DCN proteins (DCN, CAR-DCN, *m*CAR-DCN from left to right) were expressed in mammalian cells, purified on a chromatography, separated on gradient SDS/PAGE gels, and detected with a monoclonal anti–6-histidine tag antibody. The recombinant DCNs migrate as sharp bands at 45 kDa with a smear above the sharp band. The sharp bands correspond to the core proteins and the smear is caused by the chondroitin sulfate chain attached to DCN molecules. 0.5 µg of recombinant protein was loaded on each well. **(B)** Recombinant decorins (CAR-DCN, lanes # 1 and # 2 and DCN, lanes # 3 and # 4) were ran on SDS/PAGE gel right after the chromatography purification and the gel was Coomassie-stained. Both CAR-DCN and DCN were tested on non-reducing and reducing (1 µl DTT in loading buffer) conditions. 2 µg of recombinant proteins was loaded on each well. **(C)** CAR-DCN binds to heparan sulfate. On an Octet System, an Octet biosensor was coupled to heparan sulfate oligosaccharides. As a reference control, lactose was coupled to a probe using the same chemistry. Proteins were applied at different molarities (nM) in a physiological buffer, then allowed to dissociate. Reference lactose sensor binding were subtracted. Association/dissociation curves for CAR-DCN and DCN are presented. While increasing binding ability to heparin sulfate was detected with increasing protein molarities of CAR-DCN, no binding was detected for DCN.

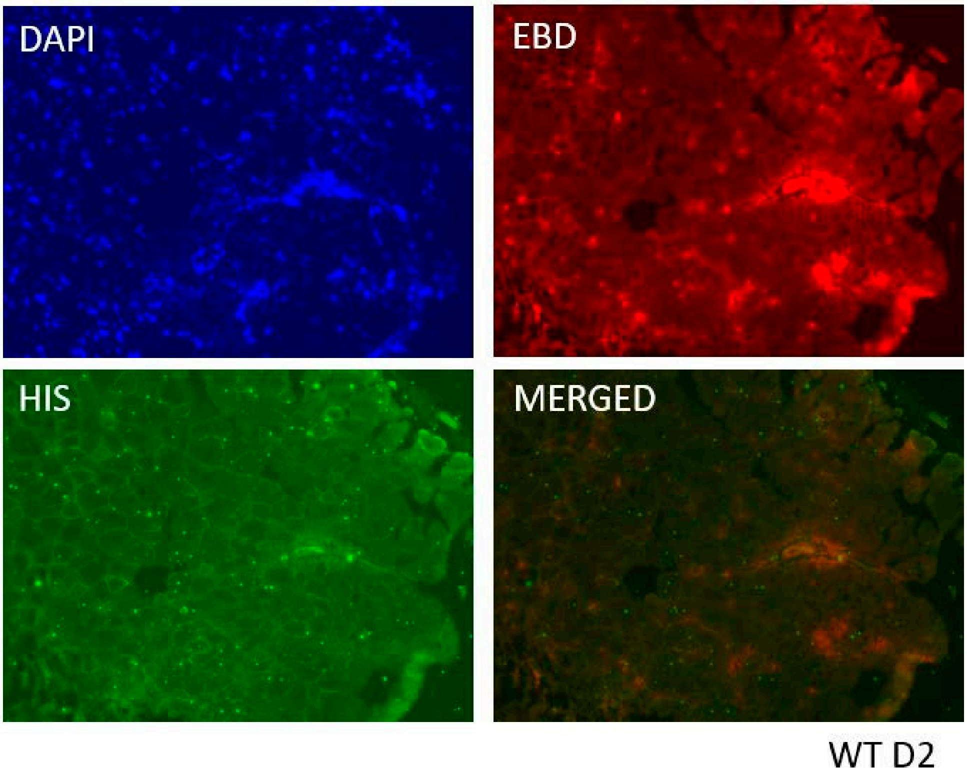

Figure S2. Three weeks of CAR-DCN treatment did not cause protein localization in wild type muscle tissues. Representative images of wildtype D2 quadriceps after three-week alternate days IP injections of CAR-DCN joined to the 6xhistidine tag. Immunofluorescence with anti-histidine tag antibodies revealed no staining in the muscle tissue.

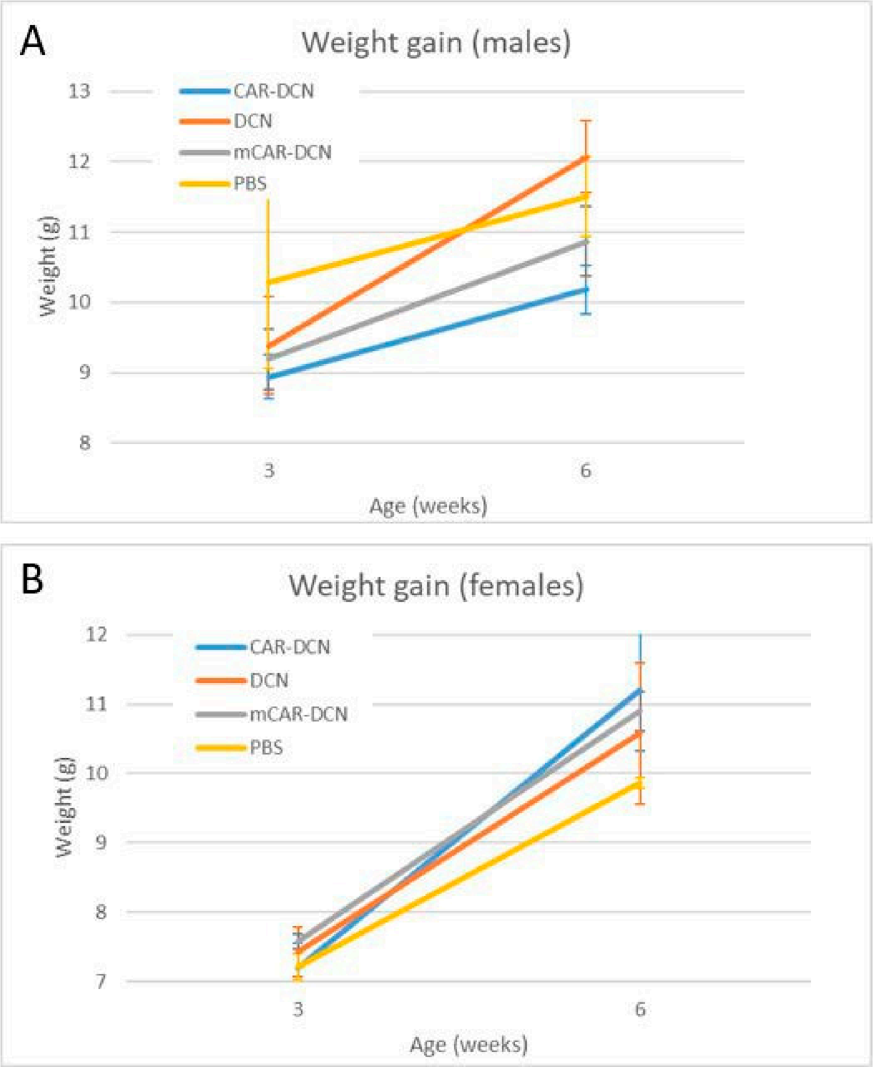

Figure S3. None of the treatments affected the weight gain of the animals over the three-week treatment course. All animals were weighted at the beginning and at the end of the treatment protocols. None of the animal cohorts' weight gain was significantly different from the other groups.

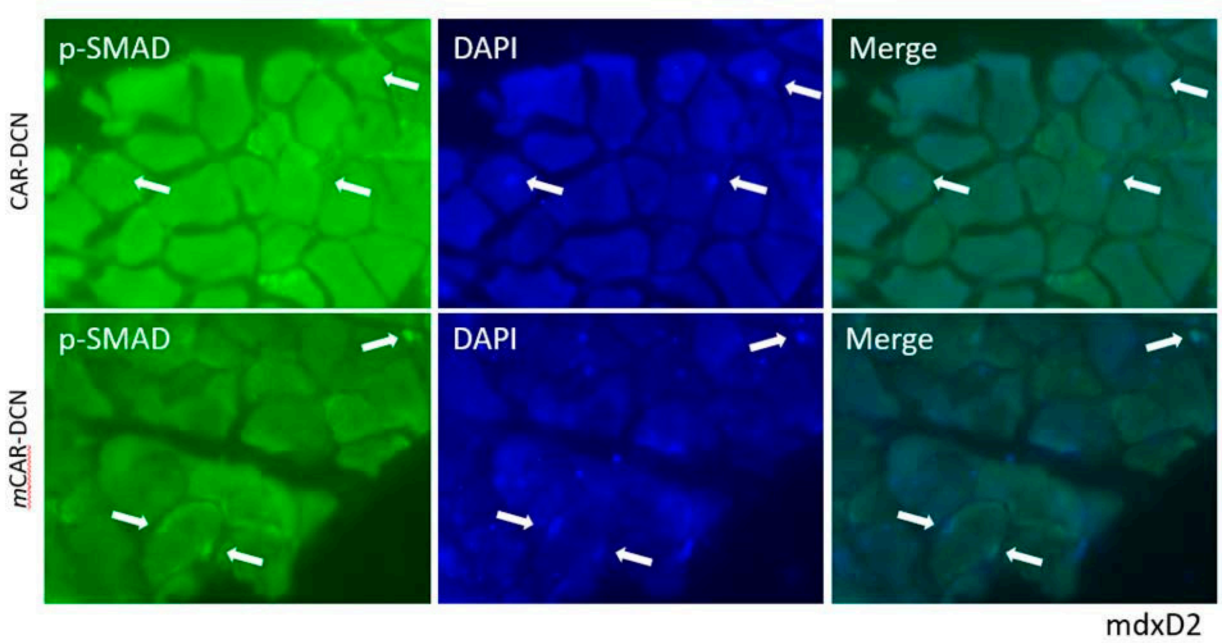

Figure S4. CAR-DCN reduces p-SMAD2/3 nuclear staining in mdxD2 quadriceps muscles. Mice were systemically treated with CAR-DCN or *m*CAR-DCN for three weeks. Nuclear p-SMAD2/3 was detected with a specific primary antibody (first column green staining). p-SMAD2/3 was not visible in the CAR-DCN treated muscle cell nuclei (arrows, top row). In the three-week *m*CAR-DCN treated mice, p- SMAD2/3 was visible in multiple nuclei (arrows, bottom row). The second column shows nuclei stained with DAPI and the final column merges the two colors to verify the nuclear localization of the p– SMAD2/3 in the *m*CAR-DCN treated mice (bottom row).

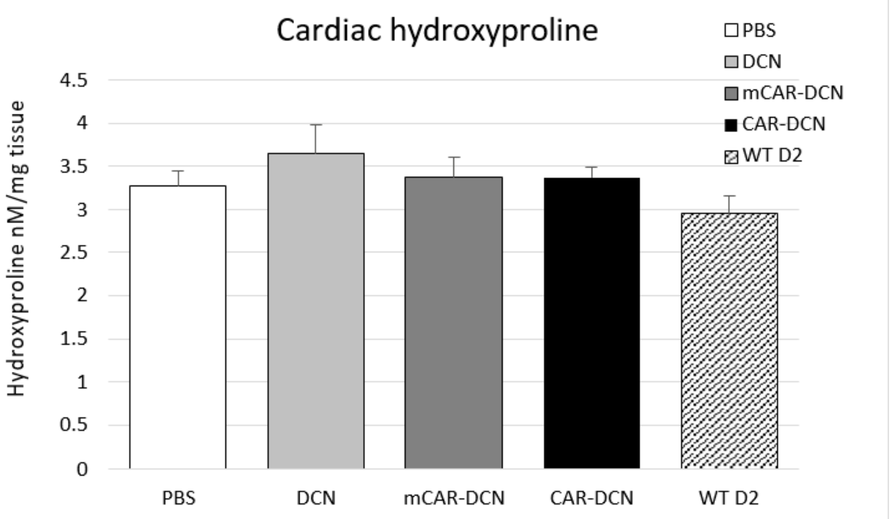

Figure S5. At six weeks old the mdxD2 mice did not have significantly more fibrosis than the wild type control mice. Therefore, none of the treatments demonstrated any changes to fibrosis.

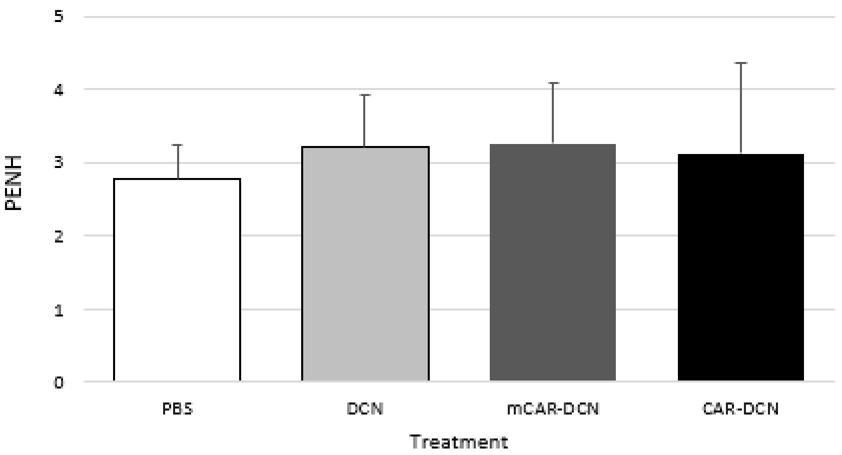

Figure S6. Three weeks of CAR-DCN treatment did not affect any of the respiratory parameters of the SgcgD2 mice. Two days before euthanasia the mice were analyzed via a whole-body plethysmography apparatus. PENH is shown as a representative of the respiratory values analyzed.

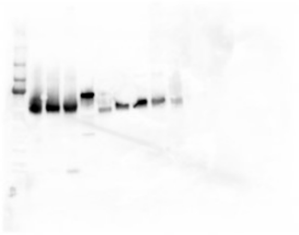

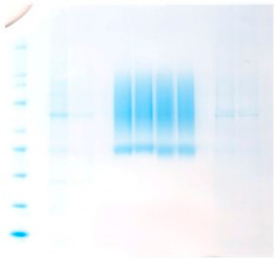

Figure S7. Uncropped western blot membrane and Coomassie-stained SDS/PAGE-gel. The membrane and the gel are for supplementary Figure S1. Western blot membrane has additional recombinant proteins (purified by 6-histidine tag) that are not related to study.
